# The masseter-buccinator periosteal flap prior to vertical bone augmentation in the mandibular region: case report and technical notes

**DOI:** 10.1186/s40729-026-00699-x

**Published:** 2026-07-02

**Authors:** Elias Jean-Jacques Khoury, Tristan Hampe, Fouad Khoury

**Affiliations:** 1 Private Clinic Schloss Schellenstein, Olsberg, Germany; 2 Department of Oral and Maxillofacial Surgery, University of Münster, Münster, Germany

**Keywords:** Pedicled masseter-buccinator periosteal flap, 3D bone augmentation, Hard- and soft-tissue reconstruction, Autogenous bone graft, Split bone block technique

## Abstract

**Background:**

Vertical bone augmentation in the posterior mandible presents significant challenges due to anatomical constraints and frequent deficiencies in soft tissue. Optimizing the soft-tissue phenotype prior to augmentation is critical to ensure stable wound closure and to provide the vascular support necessary for successful graft integration. This article introduces the pedicled masseter-buccinator periosteal flap, a novel technique that uses highly vascularized tissue to improve the quality and quantity of soft-tissue covering while also promoting graft survival.

**Case presentation:**

A patient presented with an edentulous site in the left mandibular molar region, exhibiting a severe combined vertical and horizontal ridge deficiency. Panoramic radiography revealed a non-restorable implant and a hopeless tooth in the premolar area, resulting in an extensive defect at this site. Following removal of the implant and tooth, a large three-dimensional ridge defect was confirmed intraoperatively. After local decontamination, a pedicled masseter-buccinator periosteal flap was dissected from the inner aspect of the cheek. A partial-thickness flap, which included limited buccinator and masseter muscle fibers along with the periosteal layer was elevated. The flap was rotated mesially and sutured to the lingual periosteum to achieve stable soft tissue augmentation. Finally, the overlying primary mucoperiosteal flap was sutured, resulting in a double-layered tissue closure. Two months later, three-dimensional ridge augmentation in the left mandibular premolar-molar region was performed using the split-bone block technique with autogenous graft harvested from the ipsilateral mandibular retromolar area. After three months, two implants were inserted in combination with a Kazanjian vestibuloplasty. After three months of healing, the implants were uncovered, allowing completion of the prosthetic rehabilitation. Radiographic follow-up at two years showed stable peri-implant bone levels and clinical examination confirmed healthy and stable soft-tissue conditions.

**Conclusion:**

The pedicled masseter-buccinator periosteal flap demonstrated to be a reliable and effective approach for soft tissue augmentation in the posterior mandible and support vertical bone grafting. Early clinical results indicate a high level of predictability with few complications making this technique a promising option in anatomically challenging cases.

**Graphical abstract:**

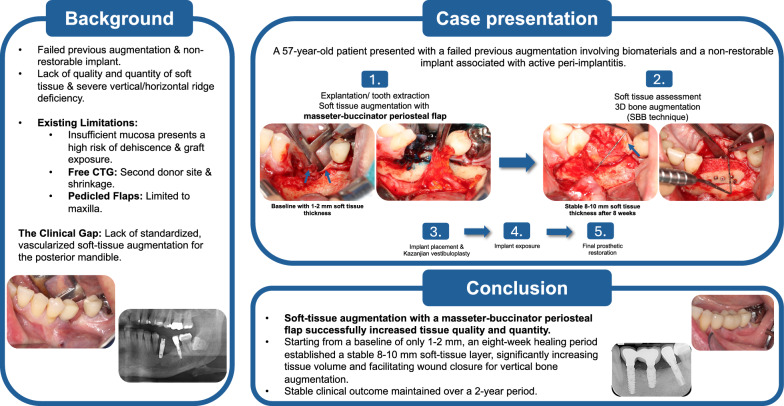

## Background

Vertical bone augmentation in the posterior mandible is highly demanding and technique-sensitive [1] due to anatomical constraints like restricted vestibular depth, thin lingual mucosa, mental nerve proximity and high muscle activity [2]. Compounding these anatomically related difficulties, vertical augmentations are often necessitated by failed prior procedures, leading to scar tissue and decreased soft-tissue quality [3, 4]. However, the attainment of successful clinical outcomes is contingent upon sufficient soft-tissue quantity and quality, as these factors determine the ability to achieve tension-free closure, protect the graft, and support uneventful wound healing [5–7].

To optimize soft-tissue quality and volume prior to large augmentation procedures, a variety of techniques have been described [1, 8]. Although free connective-tissue grafts are frequently employed, they require a second donor site and are susceptible to postoperative shrinkage due to their limited intrinsic vascularity [9–13]. Although pedicled palatal flaps provide superior vascularization, their limited rotational arc restricts their use to the maxilla [1, 14, 15]. Consequently, a standardized and dependable technique for vascularized soft-tissue reconstruction in the posterior mandible remains unavailable.

Transposing the concept of a pedicled flap to the posterior mandible introduces further anatomical complexities. Lingual flap advancement is restricted by critical neurovascular structures, including the lingual nerve submandibular duct, as well as by the intricate muscular anatomy of the floor of the mouth [16, 17]. In contrast, the posterior buccal region offers a substantial reservoir of soft tissue due to its thick, well-vascularized multilayer architecture [18]. A regional transition is evident in the cheek’s structure: the premolar region is characterized by a relatively thin wall, consisting primarily of the buccinator muscle [19]. Progressing posteriorly toward the molar and retromolar regions, the tissue becomes increasingly voluminous as the buccinator is reinforced by the buccal fat pad and the overlapping fibers of the superficial masseter [18–23]. This regional shift from a thin anterior wall to a robust, multilayered posterior configuration provides the requisite tissue bulk and reliable perfusion supplied mainly by branches of the facial and buccal arteries [20–24].

These anatomical advantages have long been utilized in maxillofacial reconstructive surgery, as a reliable donor site for local flap reconstruction [18, 24–26]. Because they combine a short arc of rotation with a thick, mechanically stable tissue block and a strong vascular pedicle, regional muscle and myomucosal flaps, such as the masseter muscle flap and buccinator-based island flaps, have been effectively used for intraoral defect coverage [21, 27, 28].

Drawing on the principles of established flap designs, a conceptual modification can be proposed: a pedicled composite flap that combines buccinator, masseter, and periosteal tissues. This composite flap yields a notably thick and well-vascularized soft-tissue segment, ideally suited for double-layer closure, where the periosteal layer reinforces the augmentation space and the vascularized mucosa ensures durable coverage. The pedicled masseter-buccinator periosteal flap thus presents a novel approach for managing severe soft-tissue deficiencies prior to vertical ridge augmentation and eliminates the need for a palatal donor site and thereby significantly reduces the risk of necrosis.

To the best of our knowledge, this specific technique has not previously been described in the literature. The present case report documents its first clinical application and illustrates its potential to establish a stable, vascularized soft-tissue foundation for complex bone augmentation in the posterior mandible.

## Case presentation

### Patient information

A 57-year-old non-smoking female patient presented with a history of a previously failed vertical ridge augmentation in the left posterior mandible, which had been performed using a xenogenic bone substitute material. Ethics approval was not required according to the institutional ethics committee, and written informed consent for publication was obtained from the patient.

Clinical examination revealed suppuration upon probing around the implant in the left mandibular second premolar region, indicative of active peri-implant infection. Pus discharge was observed, bleeding on probing was positive (BOP +), and the mean peri-implant probing depth measured 7 mm ± 0.5 mm across six sites—findings consistent with peri-implant disease. The patient reported compromised masticatory function but exhibited no acute systemic symptoms. Her medical history was unremarkable, with no systemic conditions identified that could adversely affect bone or soft tissue healing. The patient had not participated in a structured periodontal maintenance program in previous years, resulting in the progressive loss of several teeth.

### Clinical findings and diagnostic assessment

**Fig. 1:**
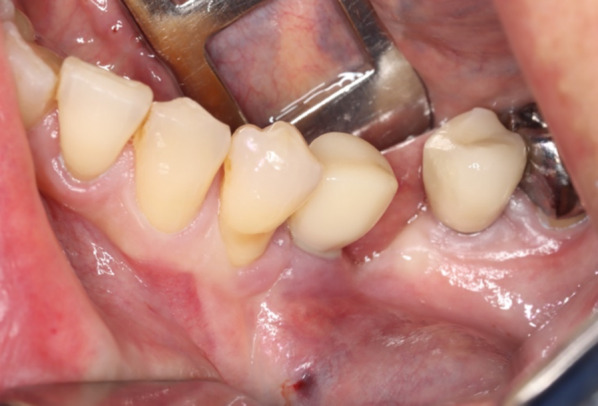
Initial presentation: large combined vertical and horizontal bone and soft tissue defect in the left mandibular region

**Fig. 2:**
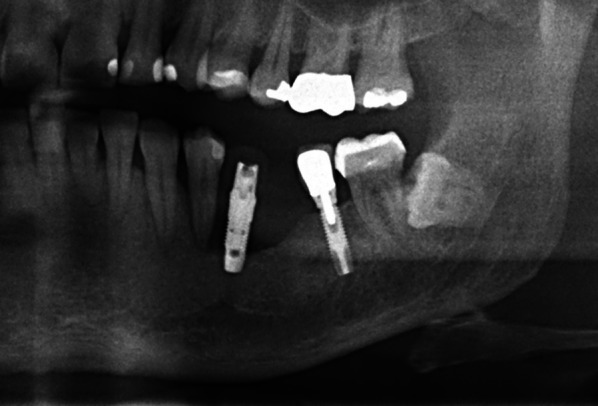
Panoramic x-ray shows marked vertical alveolar ridge deficiency and a non-restorable implant in the left mandibular region

Clinical examination revealed a pronounced combined vertical and horizontal hard- and soft-tissue defect in the left mandibular region (FDI notation: region 34–36) accompanied by a shallow vestibule and insufficient keratinized mucosa (Fig. 1). Panoramic radiography revealed a pronounced vertical ridge deficiency, a non-restorable implant in the left mandibular second premolar region, and severe periodontal breakdown of the adjacent first premolar caused by infection originating from the failing implant site (Fig. 2). As a result, both the implant and the tooth were considered non-salvageable and indicated for removal.

### Surgical procedures and technical notes

All surgical procedures were performed by a single experienced surgeon (F.K.) to ensure procedural consistency.

The patient underwent preoperative professional oral hygiene, including ultrasonic debridement, oral hygiene instruction, and a two-minute rinse with 0.2% chlorhexidine mouthwash. Perioperative antibiotic prophylaxis consisted of intravenous penicillin G (1,000,000 IU). All procedures were performed under local anesthesia (articaine 4% with 1:100,000 epinephrine; Ultracain D-S forte, Sanofi, Paris, France) in combination with conscious sedation using midazolam (5 mg/5 ml; Hameln, Germany).

**Fig. 3:**
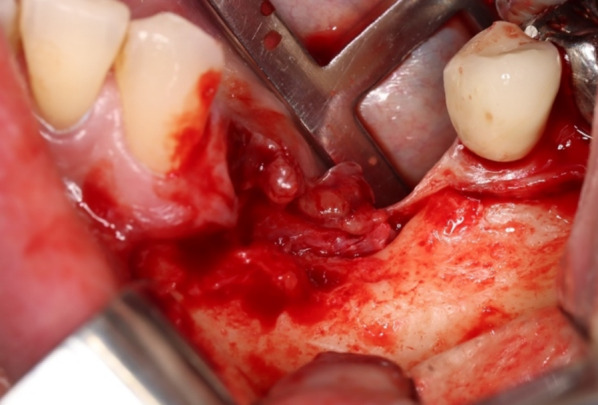
Intraoperative findings following implant explantation and tooth extraction, revealing an extensive alveolar ridge defect and a baseline soft tissue thickness of only 1–2 mm

The non-restorable implant in the left mandibular second premolar position was atraumatically explanted with the BTI system (Alava, Spain), and the adjacent first premolar was extracted (Fig. 3). The baseline situation presented a challenge, with a site-dependent vertical soft-tissue thickness of only 1–2 mm, which necessitated improvement to establish a more resilient foundation. A full-thickness mucoperiosteal flap was first raised to expose the alveolar defect. The site was then irrigated with 3% hydrogen peroxide and decontaminated using photodynamic therapy (Helbo®, Bredent, Senden, Germany). A horizontal mucosal incision was made with a 15C blade, followed by dissection through the mucosa and submucosa while including a thin layer of buccinator muscle fibers. In the molar region, where buccinator fibers predominantly run horizontally, the flap was elevated as a partial-thickness unit comprising mucosa, submucosa, limited buccinator fibers, and the underlying periosteum. In the posterior molar and retromolar region, where the anterior border of the masseter is near the buccinator muscle, a small portion of superficial masseter fibers was deliberately incorporated into the flap. This posterior muscular contribution increased flap robustness without adding excessive bulk. Throughout the dissection, the buccal fat pad was carefully preserved, and the deep masseteric layer and parotideomasseteric fascia were left intact to maintain flap pliability and minimize donor-site morbidity.

**Fig. 4:**
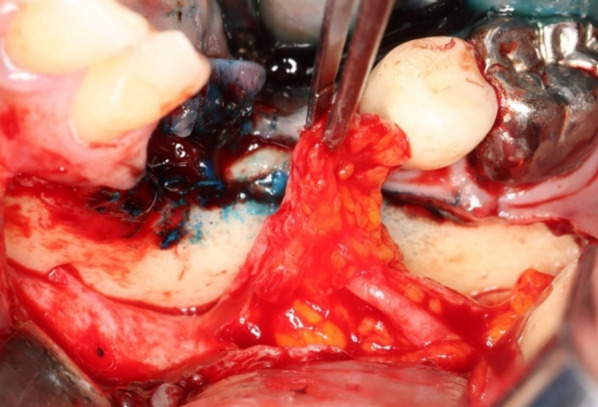
Following H₂O₂ rinse and photodynamic decontamination, a masseter-buccinator periosteal flap was elevated

**Fig. 5:**
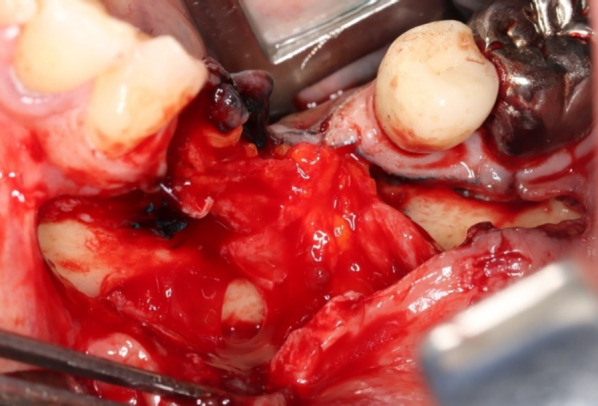
The flap was mobilized to cover the defect and sutured to the lingual periosteum to ensure stable adaptation

**Fig. 6:**
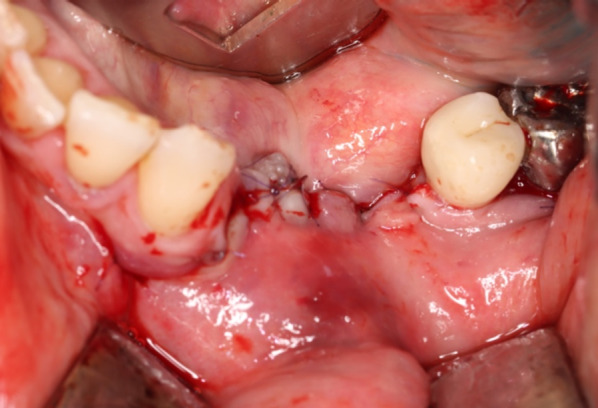
Bilayer closure with monofilament 6–0 suture

The flap was mobilized by means of blunt and sharp dissection, while maintaining its vestibular pedicle to maintain perfusion via branches of the facial and buccal arteries (Fig. 4). To guarantee stable coverage, the flap was rotated over the defect site after reaching sufficient mobility and sutured to the lingual periosteum (Fig. 5). Final bilayer closure was accomplished using 6–0 absorbable monofilament sutures (Glycolon 6–0, Resorba, Nuernberg, Germany) (Fig. 6). All remaining dentitions underwent full-mouth scaling and root planing during the surgical visit.

**Fig. 7:**
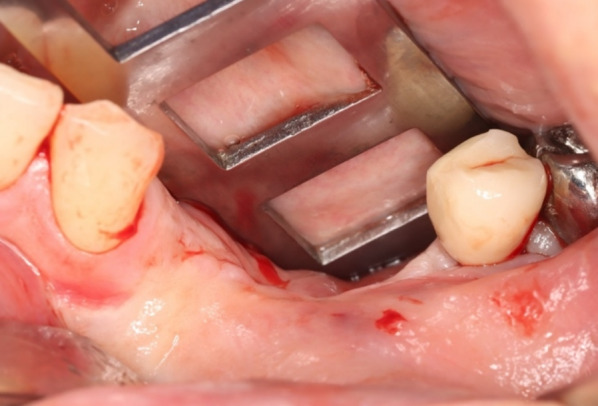
Following an eight-week healing phase, the soft-tissue phenotype was substantially improved

**Fig. 8:**
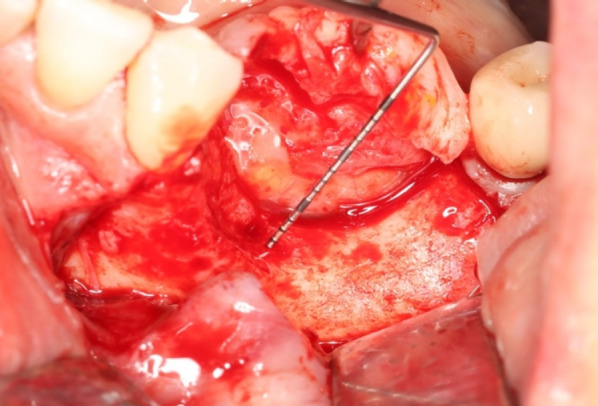
Soft-tissue assessment showing a significant increase in vertical thickness (8–10 mm)

**Fig. 9:**
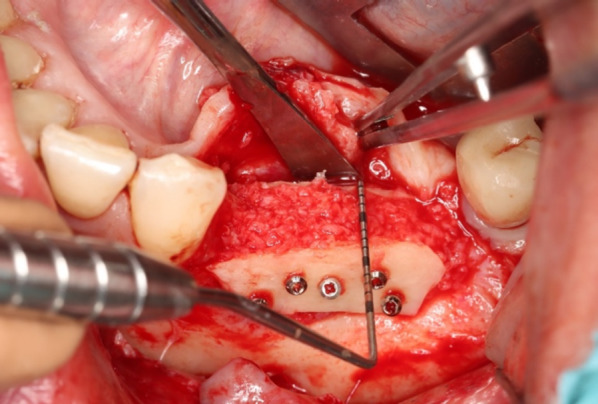
3D bone augmentation in the left mandibular region using the split-bone block technique, with autogenous graft harvested from the ipsilateral mandible; lingual mucosa showing a significant increase in soft tissue thickness

Antibiotic therapy was continued for five days postoperatively. The patient was prescribed analgesics (Ibuprofen 400 mg) and regular mouth rinses (chlorhexidine 0.2%, Chlorhexamed forte nonalcoholic 0.2%). Two weeks postoperatively, the sutures were removed following uneventful healing. The patient reported no altered function or sensibility in the region. Following an eight-week healing period, a substantial increase in soft-tissue thickness was observed (Figs. 7 and 8), with the lingual aspect in particular exhibiting a robust and well-defined soft-tissue layer reaching 8–10 mm in thickness that facilitated subsequent vertical 3D ridge augmentation. An autogenous bone block was harvested from the ipsilateral mandibular retromolar external oblique ridge using the MicroSaw (Dentsply Sirona, Charlotte, NC, USA) as described by Khoury and Hanser [29]. The graft was split according to the split-bone block technique and adapted to the defect to restore the vertical ridge dimension [1, 2, 30, 31] (Fig. 9).

**Fig. 10:**
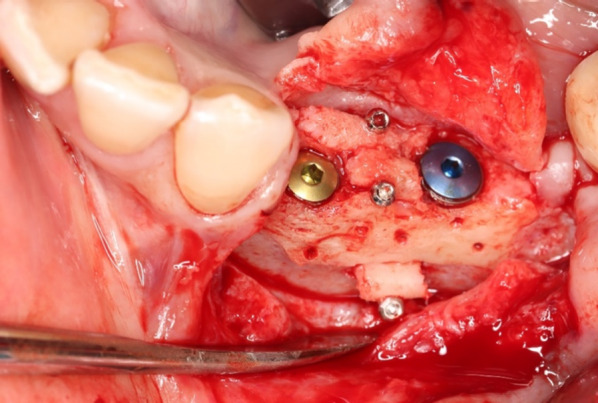
At 3 months post-op, implants were placed. Additional augmentations were performed with bone cores harvested from the implant beds

**Fig. 11:**
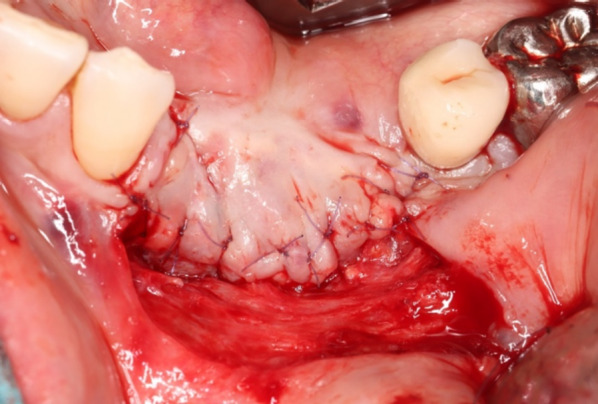
Wound closure according to the Kazanjian vestibulaplasty

**Fig. 12:**
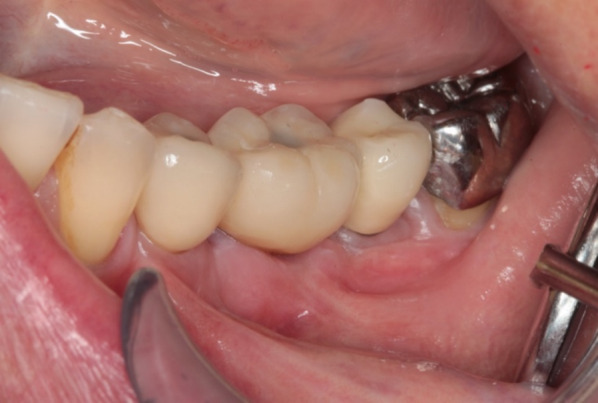
Clinical situation after prosthetic restauration

Implants were placed after a three-month graft healing period in conjunction with a Kazanjian vestibuloplasty and implant bed preparation was performed using trephine burs (Trephine-Ejection Kit, Meisinger, Neuss, Germany). The bone cores were used for minor horizontal augmentation and Implant bed preparation was continued using the implant system drills [32] (Fig. 10). The Kazanjian vestibuloplasty approach employed during implant placement enabled simultaneous soft-tissue optimization, resulting in an improved peri-implant soft-tissue phenotype and enhanced vestibular depth (Fig. 11). Three months later, the implants were exposed and subsequently restored following standard prosthetic protocols (Fig. 12). Radiographs were obtained preoperatively, postoperatively, at implant placement, after final prosthetic rehabilitation, and annually during follow-up. Clinical photographs were taken throughout the treatment sequence to document soft-tissue maturation.

### Follow up

**Fig. 13:**
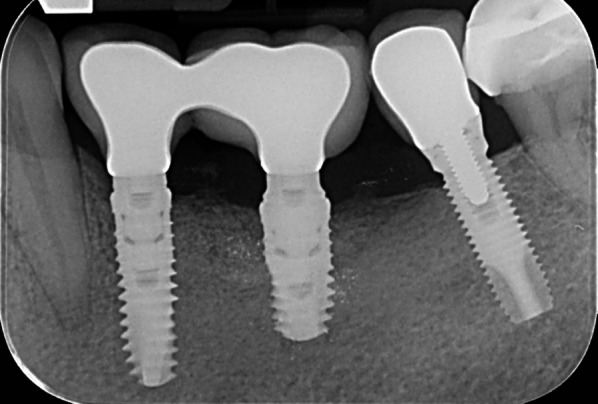
Radiographic control two years post operative

**Fig. 14:**
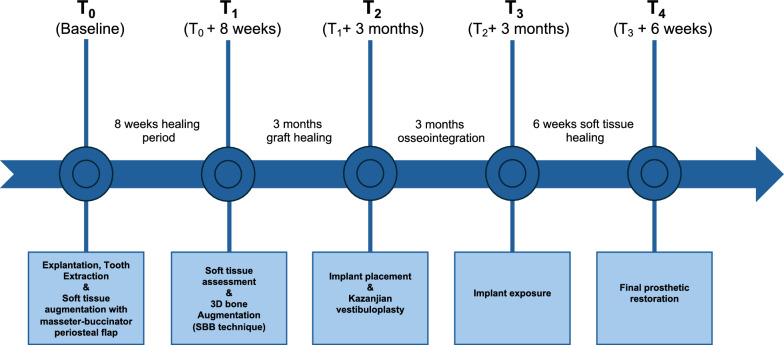
Graphical timeline illustrating the chronological sequence of the treatment steps

The patient underwent biannual follow-up visits, including professional prophylaxis and six-site periodontal and peri-implant probing. At the 2-year follow-up, the peri-implant soft and hard tissues remained healthy and stable. Clinical examination revealed no bleeding on probing, probing depths ≤ 4 mm circumferentially, and no signs of mucosal inflammation or suppuration. The peri-implant mucosa appeared firm and well-keratinized, and radiographs demonstrated stable crestal bone levels around both implants (Fig. 13). The graphical timeline illustrates the chronological sequence of the treatment steps (Fig. 14).

## Discussion

The present case demonstrates that the pedicled masseter-buccinator periosteal flap may serve as a reliable alternative to free connective-tissue grafts for soft-tissue augmentation prior to vertical ridge reconstruction in the posterior mandible. In this anatomically demanding region, which is characterized by limited vestibular depth, dense muscular attachments and the close proximity of critical structures such as the mental nerve, soft-tissue management is challenging. These difficulties were further aggravated in the present case by compromised soft-tissue conditions resulting from a failed augmentation and peri-implant inflammation. Under these circumstances, the use of a vascularized pedicled masseter-buccinator flap provided stable and resilient soft tissue and enabled predictable, uneventful healing despite the complexity of the posterior mandibular site. A marked increase in soft-tissue thickness was obtained without the need for a second donor site, thereby reducing patient morbidity while improving the peri-implant soft-tissue phenotype. At the two-year follow-up, the peri-implant tissues remained healthy and stable, supporting the long-term effectiveness of this technique. This is clinically relevant because the posterior mandible presents unique anatomical challenges that substantially raise the risk of wound dehiscence and graft exposure, especially when flap mobility and perfusion are limited. In such compromised situations, the use of a free connective-tissue graft would have required an additional donor site and carried the potential risk of scar formation, which may be disadvantageous for future flap release and amplify the risk of dehiscence [33].

However, from a clinical perspective, achieving sufficient soft-tissue thickness remains a prerequisite for tension-free closure and predictable vertical augmentation. In many cases, this practical benefit outweighs the theoretical concern of scar formation, particularly in sites that have already been compromised [1]. The pedicled masseter-buccinator periosteal flap preserves its intrinsic vascularity and avoids the introduction of avascular scar tissue at the recipient site. By supplying a vascularized combination of mucosa, limited muscle fibers, and periosteum, it provides a biologically favorable soft-tissue environment for predictable wound stability and graft protection.

Pedicled palatal flaps in the maxilla offer comparable biological advantages owing to their sustained vascularity and minimal shrinkage rates, yet their application is anatomically limited to the upper jaw. The masseter-buccinator periosteal flap provides a mandibular analogue to this concept, delivering a stable, well-vascularized soft-tissue envelope in a region where no equivalent pedicled option has previously been available.

The structural arrangement of the posterior buccal soft tissues determines both the composition and mobility of the pedicled masseter-buccinator periosteal flap. In this region, the flap generally consists of the mucosa, submucosal connective tissue, portions of the buccinator muscle, and the periosteal layer, with superficial masseter fibers occasionally included [22, 23, 34]. This configuration maintains pliability while behaving as a locally pedicled flap with a consistent musculoperiosteal blood supply, rather than as a purely random-pattern graft. Perfusion is sustained through a dense local network formed by intramuscular branches of the buccal and facial arteries [22, 26]. Careful dissection that preserves the periosteal microvasculature of the mandibular body is essential for maintaining flap viability and postoperative stability [22]. Although this dense vascularization supports reliable flap survival, it also requires precise anatomical knowledge to avoid intraoperative complications. Adequate vasoconstriction is therefore essential to reduce intraoperative bleeding and maintain good visibility, particularly when muscle fibers are included.

In the premasseteric region, the blood supply is mainly provided by the premasseteric branch of the facial artery, which arises independently and courses cranially along the anterior border of the masseter muscle. This vessel usually divides into a superficial branch, found in 92.59% of cases, and a deep branch, present in 62.96% of cases [35]. The deep branch penetrates approximately 5 mm into the masseter muscle, while the superficial branch remains on the muscular fascia.

Vascular communication with the adjacent buccinator region is enabled by the vessel’s position within the buccal fat pad. Within this compartment, a dense anastomotic network develops, allowing the premasseteric branch to connect with the buccal branch of the maxillary artery in 22.22% of cases and with the transverse facial artery in 37.03% of cases [35]. The transverse facial artery provides a significant horizontal vascular connection bridging the masseteric and buccinator territories [35].

For surgical safety during flap elevation, it is crucial to note that the facial artery is situated lateral to the anterior fibers of the masseter muscle and is generally protected by the muscle belly. Nevertheless, meticulous dissection is imperative, as the artery is at considerable risk of injury, which may result in severe hemorrhage, particularly when the buccinator muscle is detached from the mandible. At the level of the second molar, the facial artery courses in close proximity to the lateral mandibular cortex. While this relationship still allows a measurable margin for periosteal dissection, the integrated anatomical network demands caution along the anterior masseteric interface to avoid vascular damage, while simultaneously offering a dependable foundation for the stable perfusion of the elevated soft tissues [22, 23, 35, 36].

In addition to vascular safety, preservation of neurological integrity is equally critical during flap mobilization. The buccal nerve (*N. buccalis*), a sensory branch of the mandibular division of the trigeminal nerve (V3), transverses the anterior border of the mandibular ramus before descending anteroinferiorly toward the buccal mucosa [37]. As clarified by Iwanaga and Tubbs [38], the buccal nerve courses superficially in the retromolar region, lateral to the buccinator muscle, thereby contradicting earlier depictions that suggested a deeper or intramuscular course. Hendy et al. [39] further demonstrated that the buccal nerve transverses a superficial plane anterior to the ramus before branching toward the buccal gingiva of the posterior mandibular teeth.

Given this anatomical pathway, the risk of iatrogenic injury during flap elevation is minimal when the dissection remains close to the periosteum and avoids deep lateral tunneling toward the ramus [34]. However, forceful posterior manipulation or extension into the retromolar region may endanger the buccal nerve [38]. Because the flap described in the present case is advanced primarily in an anteroinferior direction and remains medial to the parotideomasseteric fascia, the likelihood of disturbing the buccal nerve is low [38]. Nevertheless, precise knowledge of the nerve’s course remains essential to prevent postoperative sensory disturbances of the buccal mucosa and gingiva.

Oral and maxillofacial surgery offers several axial-pattern flap options for intraoral reconstruction and defect coverage, including the buccinator myomucosal island flap, the facial artery musculomucosal (FAMM) flap, masseter muscle flaps, and mylohyoid myofascial flaps [20, 28, 40–42]. Although these methods provide strong perfusion, they usually involve full-thickness muscle and mucosa, which leads to significant bulk and donor-site morbidity [31, 43]. These characteristics render them ideal for mucosal resurfacing [27], but less suitable for pre-implantological sites where a thin, pliable soft-tissue envelope is required rather than a voluminous mucosal replacement.

In contrast the pedicled masseter-buccinator periosteal flap described in the present case report differs fundamentally in composition, surgical plane, and biological purpose. Instead of mobilizing a full-thickness myomucosal or myofascial flap, a partial-thickness soft-tissue unit comprising of a thin mucosal layer, submucosa, limited buccinator and posterior masseter fibers, and the periosteal component was utilized. This modification results in a thinner, more adaptable flap that maintains reliable vascularization via the intramuscular and periosteal plexus [25]. By including only small portions of the buccinator and superficial masseter muscles, the flap provides sufficient structural stability for vertical augmentation without causing the functional impairment or excessive bulk characteristic of conventional reconstructive flaps.

Beyond the flap architecture described above, including the periosteal layer offers additional biological advantages. The periosteum, particularly its inner cambium layer, is rich in osteogenic and angiogenic progenitor cells. This metabolically active interface promotes neovascularization, improves early bone and soft-tissue integration, and ultimately contributes to long-term stability [43–48]. In compromised recipient sites with decreased osteogenic capacity, such as those impacted by prior graft failure or infection, maintaining this layer within the flap improves the regenerative potential [1]. Importantly, this technique can also be used during bone augmentation procedures to achieve bilayer closure, helping to minimize the risk of postoperative dehiscence. Furthermore, the pedicled masseter-buccinator periosteal flap is feasible for use in vertical bone augmentation performed via a lateral approach, expanding its applicability in complex reconstructive scenarios.

The described flap operates as a locally pedicled unit with a consistent musculoperiosteal blood supply, distinguishing it from purely random-pattern flaps. Unlike traditional axial-pattern flaps, this flap does not depend on a single dominant vessel. Instead, its perfusion is maintained by terminal branches of the facial and buccal arteries running within the muscular pedicle. In contrast to conventional myomucosal flaps used for extensive mucosal resurfacing, the masseter-buccinator periosteal flap is primarily intended to provide a vascularized protective envelope. It is specifically designed to increase soft-tissue thickness, facilitate tension-free closure, and enhance graft stability. Therefore, this method is a conservative, minimally invasive development of well-known flaps based on the buccinator and masseter muscle, tailored to the unique needs of vertical ridge augmentation in the posterior mandible. Accordingly, the pedicled masseter-buccinator periosteal flap contributes an effective alternative to free connective-tissue grafts and larger myomucosal or myofascial flaps for soft-tissue augmentation in the posterior mandible.

In the present case, this approach provided a substantial and stable increase in soft-tissue thickness without evidence of excessive scar formation during subsequent vertical augmentation. The vascularized nature of the flap, its limited donor-site morbidity, and its favorable handling characteristics make it particularly suitable for sites previously compromised by graft failure or infection.

Nevertheless, both this technique and the present case report have limitations. Anatomically, the mesial extension of the flap is constrained by the proximity of the mental nerve and the functional limitations of the mentalis muscle. Due to the absence of a defined vascular pedicle, the flap design necessitated a broader base relative to the distal end to preserve perfusion. Considering these limitations, this technique is best applied to posterior mandibular defects, while more anterior defects may still require alternative soft-tissue augmentation approaches. The flap should be regarded as a targeted, minimally invasive solution designed to address the specific anatomical challenges of the posterior mandible, offering a clinically useful and biologically driven approach to improving soft-tissue conditions prior to vertical ridge augmentation.

Despite the favorable clinical outcome, methodological limitations must be acknowledged. Standardized volumetric digital methods, like intraoral scanning or high-resolution ultrasonography, which are increasingly advised for objective evaluation, were not used to quantify soft-tissue alterations. As this is a case report, the results cannot be generalized, and direct comparison with well-established methods such as free connective-tissue grafts is limited due to the lack of a control group. Furthermore, while the 2-year follow-up is promising, longer-term data are needed to confirm the augmented phenotype's permanent stability.

To objectively assess the soft tissue results of this technique, future research should include controlled clinical studies with larger sample sizes and standardized volumetric assessments. Long-term follow-ups will also be necessary to determine its stability, predictability, and broader clinical applicability.

## Conclusion

The pedicled masseter-buccinator periosteal flap proved to be an effective technique for soft-tissue augmentation in the posterior mandible. In addition to improving soft-tissue quality and quantity, the flap created favorable conditions for successful bone grafting by ensuring stable, well-vascularized wound closure. The early clinical results were consistent and associated with few complications, suggesting that this approach may be particularly well suited for anatomically challenging posterior mandibular cases.

## Data Availability

All data generated or analyzed during this case are included in this published article. All data generated or analyzed during this case are included in this published article.

## Abbreviations

FAAM flap: Facial artery musculomucosal flap
SBB: Split bone block technique
